# An Increase in Functional Visual Loss: Another Side-Effect of the COVID-19 Pandemic?

**DOI:** 10.3390/life15050699

**Published:** 2025-04-25

**Authors:** Andrea Lembo, Ilia Bresesti, Sofia Ginelli, Irene Schiavetti, Paolo Nucci

**Affiliations:** 1 San Giuseppe-MultiMedica Hospital, University of Milan, 20122 Milan, Italy; sofiaginelli@yahoo.it (S.G.); paolo.nucci@unimi.it (P.N.); 2 Department of Medicine and Surgery, University of Insubria, 21100 Varese, Italy; ilia.bresesti@uninsubria.it; 3 Department of Health Sciences (DISSAL), University of Genova, 16132 Genova, Italy; irene.schiavetti@unige.it; 4 IRCCS Ospedale Policlinico San Martino, 16132 Genoa, Italy

**Keywords:** functional visual loss, non-organic visual loss, COVID-19

## Abstract

**Background**: Functional visual loss (FVL), also known as Non-Organic Visual Loss (NOVL), is a condition characterized by visual impairment without an identifiable organic cause. FVL has been associated with psychological distress and psychiatric disorders, factors that were significantly impacted by the COVID-19 pandemic. This study aimed to assess the incidence of FVL before and after the COVID-19 pandemic and to explore potential underlying psychosocial factors contributing to its increase. **Methods**: We conducted a retrospective observational study at the University Eye Clinic, Milan, analyzing patient records from two six-month periods: pre-pandemic (January–June 2019) and post-pandemic (January–June 2023). We included patients aged 3–80 years old and collected their demographic, clinical, and ophthalmological data. Statistical analyses compared the FVL incidence rates and symptom prevalence across both periods. **Results**: The incidence of FVL significantly increased from 4.0% pre-pandemic to 9.1% post-pandemic (*p* < 0.001). Certain symptoms, such as eye irritation and luminous scotoma, showed significant changes post-pandemic. Pediatric patients demonstrated an increased tendency towards emulation behavior. **Conclusions**: The COVID-19 pandemic appears to have influenced the prevalence and characteristics of FVL, likely due to heightened psychological distress. Further research is needed to explore the long-term trends and intervention strategies.

## 1. Introduction

The clinical manifestation of visual loss without organic findings, known as Non-Organic Visual Loss (NOVL) or functional visual loss (FVL), is a common, challenging condition for several medical specialists [1].

Ophthalmologists, neurologists, and psychiatrists deal with patients experiencing significant vision loss despite no underlying cause being found [2].

Only a few reviews have been conducted regarding NOVL/FVL, showing that recent evidence about this condition is lacking [1,3]. There is currently a paucity of data regarding the clinical presentation, diagnosis, and management of NOVL/FVL. Clinicians suspect FLV mainly when there is an inconsistency between clinical examinations and a patient’s symptoms. In addition, there is evidence that FVL is related to mental health problems [3]. Traditionally, FVL is classified as *ametropic* when the visual symptoms are associated with uncorrected or inadequately corrected refractive errors (ametropia) without any underlying neurological or organic ocular pathology that can fully explain the visual loss (i.e., documented significant uncorrected refractive errors (e.g., myopia, hyperopia, astigmatism) on cycloplegic refraction; normal or near-normal ocular examinations (including of the retina and the optic nerve) not consistent with the reported vision loss; normal electrophysiological testing (e.g., VEP testing, ERG) and neuroimaging, if performed; visual acuity that improves significantly with the appropriate optical correction or reassurance, often during the same visit; and no evidence of malingering or factitious disorder).

FVL is classified as *comorbidity-related* when visual symptoms coexist with systemic, psychiatric, or neurological conditions that are known to contribute to or exacerbate functional visual disturbances, without sufficient organic findings to explain the symptoms (the presence of a diagnosed psychiatric disorder (e.g., anxiety, depression, somatization, conversion disorder) or neurological condition (e.g., migraine, epilepsy) temporally related to the onset or course of visual symptoms; inconsistent or non-organic visual findings on examination (e.g., tubular visual fields, spiraling visual field loss); a normal or inconclusive ophthalmologic and neuroimaging workup; support of the functional nature of the symptoms from clinical tests (e.g., the mirror test, optokinetic response, the fogging test, improvements with psychological support, psychiatric treatment, or multidisciplinary care)).

During the COVID-19 pandemic, restrictions such as social distancing were put in place for a prolonged period, and consequently, an increase in mental health problems was reported in several countries [4].

In this retrospective study, we aimed to verify the impact of the COVID-19 pandemic on the onset and diagnosis of NOVL/FVL in Italy.

## 2. Materials and Methods

This retrospective observational monocentric study was conducted at the University Eye Clinic, the University of Milan, San Giuseppe Hospital, MultiMedica Group (Milan, Italy).

Data were extracted from an electronic health record system (EHR), and two cohorts of patients were selected before and after the COVID-19 pandemic over a 6-month period each (1 January 2019 to 30 June 2019 and 1 January 2023 to 30 June 2023). All patients ranging 3–80 years of age were included, regardless of whether they had normal vision (emmetropia) or any pre-existing disorders. Patients with known genetic disorders, severe chronic illnesses, and a previous certified diagnosis of major psychiatric disorders were excluded from this study.

The data included demographics, past and recent medical history, ophthalmology examinations, and orthoptic evaluations.

All patients underwent a comprehensive ophthalmological examination, with initial attention given to previous findings in their eyes and their reasons for seeking a consultation. An assessment of their craniofacial and eye regions was conducted, as was an evaluation of their intrinsic and extrinsic eye motion and direct and consensual pupil reflexes.

Following the initial assessments, the patients underwent a subjective and objective refraction evaluation, along with an examination of their anterior eye segments and fundi using a slit lamp during pharmacologically induced mydriasis. Patients without organic findings in these initial tests underwent additional examinations, such as Optical Coherence Tomography (OCT), corneal topography, and computerized visual field assessments.

Where these supplementary tests did not find any organic issues, the doctors concluded FVL in the patient. In such instances, a neuro-ophthalmologist validated the diagnosis, with specific neurological tests rarely providing further confirmation. The neuro-ophthalmologist in our unit is the specialist we call to explore the relationship between the nerve compartment and the eye when the ophthalmologist needs additional consultancy.

Demographic and clinical data (FVL category, the presence of ametropias and/or ocular comorbidities, symptoms) were collected. The symptoms considered in our analysis included the following:

- Difficulties with near vision;
- Difficulties with far vision;
- Difficulties reading the blackboard;
- Visual blur;
- Headache;
- Dizziness, sickness, or vertigo;
- A burning sensation in the eyes;
- Pain around the eyes;
- Red eye;
- Itchy eyes;
- Eye irritation;
- Foreign bodily sensations;
- Xerophthalmia;
- Wavy vision;
- Flashing lights;
- Luminous scotoma;
- Metamorphopsia;
- Floaters;
- The presence of a black bar in one’s visual field;
- A reduction in one’s visual field;
- Photophobia;
- Dots of light in one’s vision;
- Flashing and rotating circles;
- Double and triple vision.

### Statistical Analysis

Descriptive data were reported as means and standard deviations or as absolute and relative frequencies, as appropriate. The Mann–Whitney U test was utilized to assess the differences in continuous variables between groups. Associations among categorical variables were determined using the chi-squared test and Fisher’s exact test. The comparison of two rates (before and after the pandemic) was expressed as the incidence rate ratio (IRR) and the Incidence Rate Difference, with their 95% Confidence Intervals (CIs) calculated using the exact Poisson method.

A *p*-value < 0.05 was considered statistically significant.

## 3. Results

In each of the study periods considered, 3600 patients were visited. Among them, 470 individuals (144 pre-COVID-19 and 326 post-COVID-19) with positive findings for functional visual loss (FVL) were selected.

No significant difference was found between the two periods regarding age (13.5 ± 14.01 years pre-pandemic vs. 11.8 ± 10.92 years post-pandemic; *p* = 0.75) and gender distribution (63.9% female and 36.1% male patients pre-pandemic vs. 60.7% female and 39.3% male patients post-pandemic; *p* = 0.52).

There was no difference between the two periods in the frequency of the types of FVL (type 1: *p* = 0.30; type 2: *p* = 0.59; type 3: *p* = 0.29). However, significantly more cases of ametropia were detected before COVID-19 compared to post-COVID-19 (38.9% vs. 20.6%; *p* < 0.001), as were more comorbidities (17.4% vs. 9.2%; *p* = 0.011).

The main data are outlined in Table 1.

Before the COVID-19 pandemic, the incidence of FVL was 4.0% (95% CI: 3.4–4.7%). After the pandemic, there was a notable increase in FVL incidence to 9.1% (95% CI: 8.1–10.1%). The absolute difference in its incidence was 5.1% (95% CI: 3.9–6.2%; *p* < 0.001), and the incidence rate ratio was also statistically significant (2.26; 95% CI: 1.86–2.77; *p* < 0.001).

The incidence of FVL was also calculated in subgroups of patients without ametropia and without comorbidities, as a statistically significant difference was found in the frequency of these events between the two periods. We excluded patients where a correlation between COVID pandemic restrictions (e.g., the increased use of screens, etc.) and worsening myopia was clear.

When considering the subgroup of patients without ametropia, the difference between the two rates remained statistically significant (4.8%; 95% CI: 3.7–5.8%; *p* < 0.001), as did the incidence ratio (2.94; 95% CI: 2.30–3.79; *p* < 0.001).

Similarly, when including only patients without comorbidities, the difference between the two rates remained statistically significant (4.9%; 95% CI: 3.8–6.0%; *p* < 0.001), as did the incidence ratio (2.48; 95% CI: 2.00–3.10; *p* < 0.001).

No stratified analysis by age or sex was conducted, as no differences were observed for these parameters between the two periods.

Beyond incidence, our investigation highlighted differences in the frequency of symptoms between the two groups, as illustrated in Table 2. Marked disparities were evident before and after the COVID-19 pandemic.

Symptoms with notable differences included red eye (*p* = 0.014), eye irritation/foreign bodily sensations (*p* < 0.001), wavy vision (*p* = 0.016), luminous scotoma (*p* = 0.006), palpebral swelling (*p* = 0.018), and burning sensations (*p* = 0.016).

In addition, a significant percentage of the patients presented at the ophthalmology visits without symptoms, ranging from 17 to 11%, before COVID-19. This percentage dropped to 0% after the pandemic.

## 4. Discussion

Our data shows a remarkable increase in FVL cases, especially post-COVID-19. This calls for a thorough investigation into its underlying causes.

We found a significant portion of cases occurring among the youngest, who exhibited emulation behavior by seeking lens corrections to boost their self-esteem. Among adults, FVL stems from various factors, notably psychiatric disorders, likely exacerbated by pandemic-induced psychosocial changes. The mass hysteria and psychosocial modifications triggered by the pandemic likely played a significant role in this increase [5].

Historically, economic crises, social upheavals, and natural disasters have been associated with an increased incidence of psychiatric disorders [6,7]. The COVID-19 pandemic, beyond its direct impact on health structures, has been extensively documented to exert a profound influence on the mental health of populations, leading to elevated rates of depression, anxiety, stress, suicidal tendencies, post-traumatic stress disorder (PTSD), and somatization disorders. These mental health challenges were further exacerbated by the stringent social restrictions imposed by governmental institutions [8].

Prior research has established a strong connection between psychiatric disorders and FVL. Psychosocial roots contribute to 32% of FVL cases, with identifiable provoking events in 78% of such cases [9,10]. Given the current study’s location in Lombardy, the first Italian region significantly impacted by the pandemic, it is plausible to consider the enduring fear of the virus and prolonged social distancing measures as contributing factors.

Notably, studies conducted in the early stages of the pandemic in the People’s Republic of China revealed that the prevalence of anxiety and depression in the general population was approximately 8% and 15%, respectively. These figures doubled to around 13% and 22%, respectively, when individuals were aware of family members or acquaintances subjected to health restrictions. Also, approximately 40% of subjects reported psychological problems, with roughly 14% experiencing PTSD [10,11].

Given these findings, it is reasonable to infer that the above elements played a significant role in the patients included in our study. The geographical origin of our patients, coupled with the persistent fear of the virus and the prolonged impact of social distancing measures in Lombardy, likely contributed to the observed increase in the incidence of functional visual loss post-COVID-19 pandemic. This underscores the broader societal implications of the pandemic on mental health and consequently on clinical presentations, such as FVL.

A noteworthy portion of the patients in our study fell within the pediatric age group (<18 years), warranting specific considerations given the significant role of social interactions in their development. This is particularly crucial up to the age of 7 and throughout adolescence, where peer groups serve as essential dimensions for psychological development and collaboration with peers supports mental and motor growth. Many reports have widely attributed the increase in the incidence of mental health problems to the pandemic restrictions, which disrupted the simple everyday lives of children [12,13,14].

Many studies have described pediatric patients as insecure, with low self-esteem and a general distrust of the world, constantly seeking certainty [15]. Pandemic-induced emulation, characterized by the impulse to imitate the actions and behaviors of others, has become widespread. In our pediatric patients, we observed a notable effect of emulation, where children mimicked visual loss to obtain optical corrections (e.g., glasses) similar to those of their friends or siblings, aiming to enhance their self-esteem [16,17].

Beyond emulation, studies indicate that in 27% of pediatric cases of FVL, pre-existing conditions such as anxiety, depression, and attention deficit hyperactivity disorder (ADHD) are co-present. We observed stress related to study activities in 31% of cases [18].

It is also crucial to address masking, particularly in children. Researchers have extensively studied this age group because they often make fewer effective attempts to conceal their symptoms [19]. Conversely, some adults may exploit claimed symptoms to seek financial compensation. Understanding these dynamics is essential for a comprehensive interpretation of the observed trends in the incidence of functional visual loss, especially among pediatric patients, emphasizing the intricate interplay of psychological and social factors in their presentation.

### Potential Developments

The observed increase in functional visual loss (FVL) may have been influenced by societal trends of chasing ideals of perfection across various aspects of life, such as work, health, and physical appearance. This pursuit of perfection, which is prevalent in modern society, has known negative implications for mental health [20]. Consequently, it is essential to consider that the rise in FVL cases could be in part because of this societal trend.

This drive for performance and perfection extends to young people, where parental pressure in terms of academic achievement and involvement in many extracurricular activities may contribute to a sense of distance from loved ones. In extreme cases, this distance can lead to attention-seeking behaviors, with potential implications for mental health, forming the foundation for FVL [21].

The role of social media, particularly in the lives of teenagers, is noteworthy. Social media content can foster conformism, where individuals strive to meet expectations and follow popular trends to gain acceptance from their peers. This conformity causes a lack of individuality and self-esteem [22]. Therefore, it is plausible to hypothesize that the incidence of FVL may continue to grow with generational changes, particularly among so-called “digital natives”. Future research could explore these societal dynamics and their impact on the developing landscape of FVL.

This study has some limitations. First is the use of a non-random sampling method, which may have introduced selection bias and affected the generalizability of the findings. Since the participants were not selected through probabilistic methods, there is a possibility that specific subgroups—such as individuals more likely to seek ophthalmologic care or those more affected by psychosocial stressors—were overrepresented in the sample. This could have led to the overestimation of the prevalence of functional visual loss (FVL) and may particularly have impacted the post-COVID-19 increase in cases observed. While our findings remain clinically relevant and reflect real-world data from a referral center, caution is warranted when extrapolating these results to broader populations. Future studies employing random or population-based sampling would be valuable to confirm and refine these observations. Also, the absence of a longitudinal follow-up is worth addressing. A lack of scheduled check-ups for the patients prevented the collection of developing trends in their symptoms and the re-examination of the patients after a diagnosis of FVL. Then, the inclusion of patients with pre-existing eye comorbidities may have led to an accentuation of symptoms and complaints in patients with pre-existing conditions. Also, it is crucial to acknowledge the differences between the incidence of FVL in neuro-ophthalmology clinics and conventional ophthalmology clinics. This study specifically considered FVL incidence in the latter setting.

Regardless of these limitations, our findings highlight an increase in FVL cases after the COVID-19 pandemic and stimulate further research on this topic. We could add insights into the complexities of FVL’s incidence and presentation by adding a long-term follow-up and targeting the selected population.

## 5. Conclusions

Identifying the cause of FVL presents challenges in medical practice, as it contradicts the expectation of a visible explanation for symptoms. FVL poses a unique challenge, as it contradicts this assumption, and the lack of a gold-standard diagnostic workflow complicates matters.

Our study sheds light on a relatively obscure and poorly defined condition known as FVL while also aiding in its diagnosis by highlighting the primary symptoms exhibited by our patients.

The convergence of epidemiological data, psychological research, and the clinical presentation of our patient population supports the notion that the relationship between the pandemic and the observed increase in FVL is not due to random variation but rather reflects a complex interplay of societal and individual factors triggered by this health crisis.

## Figures and Tables

**Table 1:** Demographic and baseline characteristics. Data are presented as absolute number (N) and percentage (%). * indicates statistical significance.

		Pre-COVID-19	Post-COVID-19	*p*
Gender	Female	92 (63.9%)	198 (60.7%)	0.52
	Male	52 (36.1%)	128 (39.3%)	
Age, years		13.5 ± 14.01	11.8 ± 10.92	0.75
Age class	3–8 years	66 (45.8%)	158 (48.5%)	0.60
	8–17 years	53 (36.8%)	123 (37.7%)	
	≥18 years	25 (17.4%)	45 (13.8%)	
*Ametropic*	Absent	88 (61.1%)	259 (79.4%)	<0.001 *
	Present	56 (38.9%)	67 (20.6%)	
*Comorbidities (conjunctival hyperemia, refractive disorders)*	Absent	119 (82.6%)	296 (90.8%)	0.011 *
	Present	25 (17.4%)	30 (9.2%)	
Type 1: hysterics	Absent	51 (35.4%)	132 (40.5%)	0.30
	Present	93 (64.6%)	194 (59.5%)	
Type 2: intentional simulators	Absent	142 (98.6%)	324 (99.4%)	0.59
	Present	2 (1.4%)	2 (0.6%)	
Type 3: subjects who exaggerate their symptoms	Absent	94 (65.3%)	196 (60.1%)	0.29
	Present	50 (34.7%)	130 (39.9%)	

**Table 2:** Differences in the symptomatology between patients examined before and after the COVID-19 pandemic. Data are presented as absolute number (N) and percentage (%). * indicates statistical significance.

		Pre-COVID-19	Post-COVID-19	*p*
Visual difficulties pl	Absent	115 (79.9%)	253 (77.6%)	0.59
	Present	29 (20.1%)	73 (22.4%)	
Visual difficulties pv	Absent	138 (95.8%)	299 (91.7%)	0.11
	Present	6 (4.2%)	27 (8.3%)	
Symptoms	Absent	17 (11.8%)	0 (0.0%)	<0.001 *
	Present	127 (88.2%)	326 (100.0%)	
Difficulty seeing the blackboard	Absent	135 (93.8%)	305 (93.6%)	0.94
	Present	9 (6.3%)	21 (6.4%)	
Headache	Absent	135 (93.8%)	306 (93.9%)	0.96
	Present	9 (6.3%)	20 (6.1%)	
*Red eye*	Absent	136 (94.4%)	321 (98.5%)	0.014 *
	Present	8 (5.6%)	5 (1.5%)	
Itching	Absent	137 (95.1%)	318 (97.5%)	0.17
	Present	7 (4.9%)	8 (2.5%)	
*Eye irritation/the sensation of the presence of a foreign body in one’s eye*	Absent	120 (83.3%)	306 (93.9%)	<0.001 *
	Present	24 (16.7%)	20 (6.1%)	
*Wavy vision*	Absent	140 (97.2%)	325 (99.7%)	0.016 *
	Present	4 (2.8%)	1 (0.3%)	
Flashing lights	Absent	137 (95.1%)	311 (95.4%)	0.90
	Present	7 (4.9%)	15 (4.6%)	
*Luminous scotoma*	Absent	136 (94.4%)	322 (98.8%)	0.006 *
	Present	8 (5.6%)	4 (1.2%)	
Metamorphopsia	Absent	141 (97.9%)	322 (98.8%)	0.48
	Present	3 (2.1%)	4 (1.2%)	
*Eyelid swelling*	Absent	138 (95.8%)	323 (99.1%)	0.018 *
	Present	6 (4.2%)	3 (0.9%)	
A reduction in one’s visual field	Absent	135 (93.8%)	311 (95.4%)	0.45
	Present	9 (6.3%)	15 (4.6%)	
Photophobia	Absent	138 (95.8%)	318 (97.5%)	0.31
	Present	6 (4.2%)	8 (2.5%)	
Bright dots	Absent	141 (97.9%)	322 (98.8%)	0.48
	Present	3 (2.1%)	4 (1.2%)	
Eye dryness	Absent	138 (95.8%)	313 (96.0%)	0.93
	Present	6 (4.2%)	13 (4.0%)	
Eye floaters	Absent	139 (96.5%)	318 (97.5%)	0.54
	Present	5 (3.5%)	8 (2.5%)	
Flashing and rotating circles	Absent	142 (98.6%)	321 (98.8%)	0.99
	Present	2 (1.4%)	4 (1.2%)	
Visual blurring	Absent	141 (97.9%)	316 (96.9%)	0.55
	Present	3 (2.1%)	10 (3.1%)	
Dizziness, nausea, and vertigo	Absent	142 (98.6%)	316 (96.9%)	0.29
	Present	2 (1.4%)	10 (3.1%)	
Diplopia or triple vision	Absent	141 (97.9%)	319 (97.9%)	0.97
	Present	3 (2.1%)	7 (2.1%)	
*Burning sensations*	Absent	140 (97.2%)	325 (99.7%)	0.016 *
	Present	4 (2.8%)	1 (0.3%)	
Periocular pain	Absent	140 (97.2%)	323 (99.1%)	0.13
	Present	4 (2.8%)	3 (0.9%)	
An altered perception of color	Absent	141 (97.9%)	322 (98.8%)	0.48
	Present	3 (2.1%)	4 (1.2%)	
Frequent blinking	Absent	139 (96.5%)	314 (96.3%)	0.91
	Present	5 (3.5%)	12 (3.7%)	

## Data Availability

The data can be made available upon reasonable request due to (specify the reason for the restriction).

## Abbreviations

The following abbreviations are used in this manuscript:

COVID-19	COronaVIrus Disease-19
FVL	Functional Visual Loss
NOVL	Non-Organic Visual Loss
EHR	Electronic Health Record
OCT	Optical Coherence Tomography
SPSS	Statistical Product and Service Solutions
IRR	Incidence Rate Ratio
CI	Confidence Interval
PTSD	Post-Traumatic Stress Disorder
ADHD	Attention Deficit Hyperactivity Disorder
